# Defective viral genomes from chikungunya virus are broad-spectrum antivirals and prevent virus dissemination in mosquitoes

**DOI:** 10.1371/journal.ppat.1009110

**Published:** 2021-02-08

**Authors:** Laura I. Levi, Veronica V. Rezelj, Annabelle Henrion-Lacritick, Diana Erazo, J Boussier, Thomas Vallet, Veronika Bernhauerová, Yasutsugu Suzuki, Lucia Carrau, James Weger-Lucarelli, Maria-Carla Saleh, Marco Vignuzzi

**Affiliations:** 1 Institut Pasteur, Viral Populations and Pathogenesis Unit, CNRS UMR 3569, Paris, France; 2 École doctorale BioSPC, Université Paris Diderot, Sorbonne Paris Cité, Paris, France; 3 Institut Pasteur, Viruses and RNAi Unit, CNRS UMR 3569, Paris, France; 4 École doctorale Frontières du vivant, Université Paris Diderot, Paris, France; 5 Department of Biophysics and Physical Chemistry, Faculty of Pharmacy, Charles University, Hradec Králové, Czech Republic; 6 Department of Biomedical Sciences and Pathobiology, Virginia Tech, VA-MD Regional College of Veterinary Medicine, Blacksburg, Virginia, United States of America; University of Michigan, UNITED STATES

## Abstract

Defective viral genomes (DVGs) are truncated and/or rearranged viral genomes produced during virus replication. Described in many RNA virus families, some of them have interfering activity on their parental virus and/or strong immunostimulatory potential, and are being considered in antiviral approaches. Chikungunya virus (CHIKV) is an alphavirus transmitted by *Aedes spp*. that infected millions of humans in the last 15 years. Here, we describe the DVGs arising during CHIKV infection *in vitro* in mammalian and mosquito cells, and *in vivo* in experimentally infected *Aedes aegypti* mosquitoes. We combined experimental and computational approaches to select DVG candidates most likely to have inhibitory activity and showed that, indeed, they strongly interfere with CHIKV replication both in mammalian and mosquito cells. We further demonstrated that some DVGs present broad-spectrum activity, inhibiting several CHIKV strains and other alphaviruses. Finally, we showed that pre-treating *Aedes aegypti* with DVGs prevented viral dissemination *in vivo*.

## Author summary

Defective viral genomes (DVGs) are produced during virus replication. On their own they cannot replicate, but some of them can compete with wild-type virus for viral and/or cellular resources. For chikungunya virus, interference by DVGs has not been described. Here, we use a new approach based on experimental evolution and computational analysis to characterize all DVGs generated in a virus population and identify those with the highest antiviral potential. We confirm their antiviral activity in both mammalian and mosquito host environments and show that some can broadly interfere with other strains or related alphaviruses. Finally, we show that DVGs can inhibit virus dissemination in mosquitoes.

## Introduction

Chikungunya virus (CHIKV) is a positive stranded RNA virus belonging to the alphavirus genus (*Togaviridae* family) that is responsible for chikungunya fever, a dengue-like syndrome with severe joint pain. This arthropod-borne virus (arbovirus) transmitted by *Aedes spp*. mosquitoes re-emerged in the last 15 years, posing a serious public health threat by causing two major worldwide epidemics with close to 8 million cases[1–3] and other frequent localized outbreaks. Its emergence in the Indian Ocean islands in 2005–2006 was caused by the Indian Ocean lineage strain (CHIKV-IOL) derived from the Eastern-Central-Southern African lineage[1–3]; while the Caribbean strain originated from the Asian lineage and caused the outbreak in the Caribbean states and the Americas in 2013–2014[2, 3]. Because there are currently no CHIKV treatment or licensed vaccines available, vector control strategy remains a main axis of prevention and outbreak control.

Along with high mutation rates, recombination is another main driving force of RNA virus evolution. Non-homologous recombination can give rise to truncated and/or rearranged viral genomes called defective viral genomes (DVGs). First described in influenza virus in 1954 by Von Magnus[4], DVGs have since been described in all viral families[5, 6], generally when virus is passaged at high multiplicity of infection (MOI) conditions that favor the appearance of defective genomes requiring helper function from full-length virus. Because they lack part of their genome or its encoded functions, DVGs must co-infect cells with their parental virus, in order to take advantage of the proteins encoded by the full-length virus. Hence, DVGs that hijack the replication machinery or use proteins encoded by the parental virus, may compete with wild-type virus for resources, which can result in inhibition of the parental virus[7, 8]. These types of DVGs are often referred to as defective interfering particles. Furthermore, many DVGs have a strong, immunostimulatory potential both in mammals and invertebrates[7, 9–11]. DVGs were identified in sera from patients suffering from acute dengue virus infection, but their pathophysiological role remains unknown[12]. In humans, their presence correlates with milder disease and better outcome in influenza virus[13] and respiratory syncytial virus infections[10]. All these reasons explain the recent renewed interest in using DVGs as antiviral therapy[14, 15].

Only a few studies cover alphavirus DVGs. In Sindbis virus, a low-fidelity polymerase was shown to recombine at higher rate, overproducing DVGs that correlated with interference[16]. In invertebrates, alphavirus DVGs (Sindbis virus and CHIKV) were shown to be a template for the viral DNA form, which is key in modulating the antiviral immune response and establishing persistent infections in insects[9]. However, no studies have focused on developing antivirals derived from alphavirus DVGs.

In this work, we document and characterize naturally occurring DVGs bearing deletions, arising during CHIKV infection *in vitro* and *in vivo*. From these, we selected the DVGs based on their frequency and recurrence between replicates and/or their ability to be carried over multiple passages. We show that natural DVGs have a strong antiviral activity on CHIKV both in mammalian and mosquito cells *in vitro*, and demonstrate that interfering DVGs can be broad-spectrum against other alphaviruses. Finally, we show that DVGs can be introduced into the mosquito vector prior to infection to inhibit arbovirus dissemination.

## Results

### Generation of defective viral genomes (DVG) by *in vitro* high MOI passage

**Fig 1:**
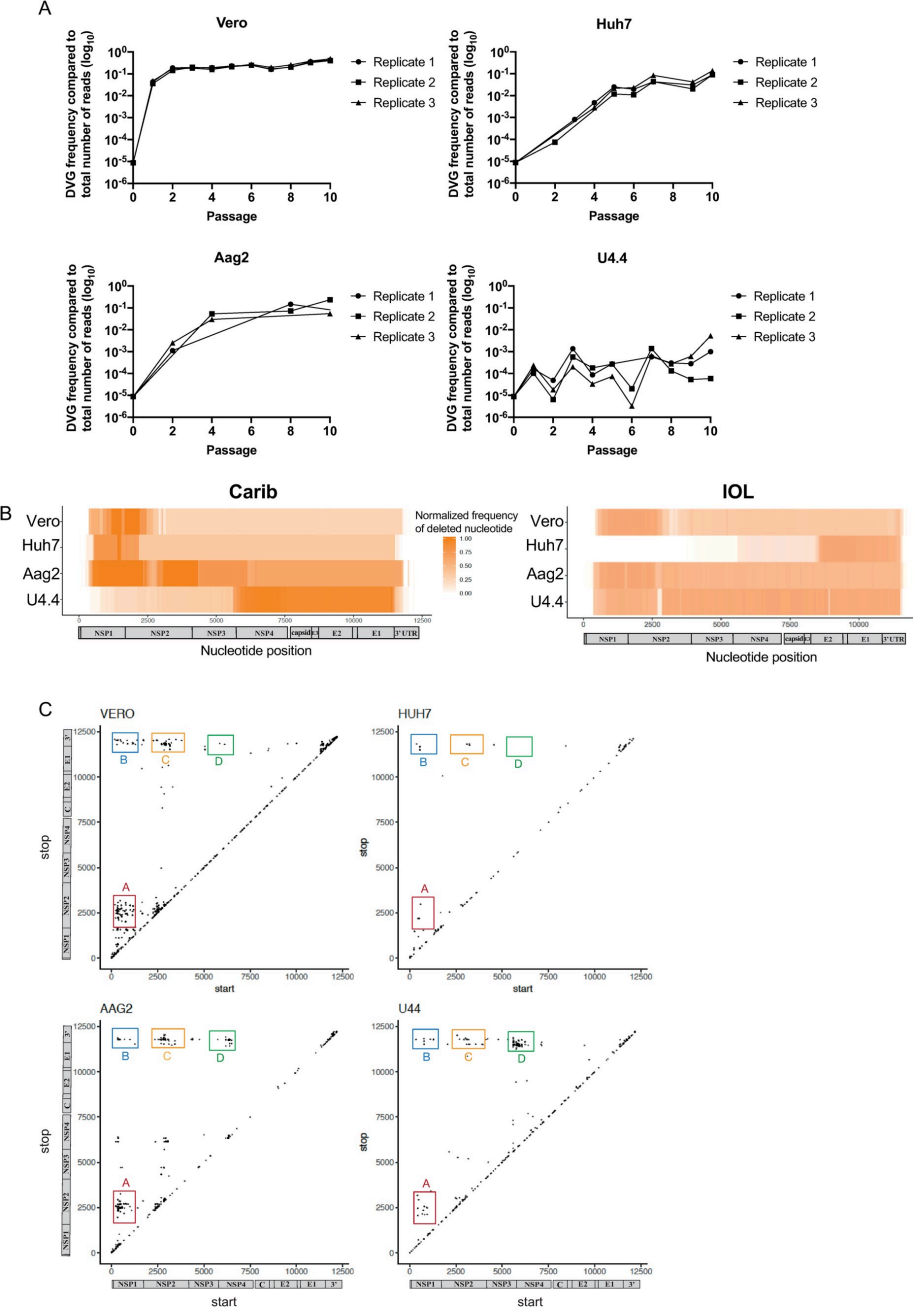
Generation of chikungunya virus (CHIKV) defective viral genomes (DVGs) in different hosts *in vitro*. The Caribbean strain (Carib) and Indian Ocean lineage (IOL) of CHIKV was passaged at high MOI in mosquito (U4.4, Aag2) or mammalian (Vero, Huh7) cells in triplicate. For each passage, the supernatant harvested from the previous passage was used to infect fresh cells. The infection lasted 48 to 72 hours. (**A**) DVG accumulation through passages. Total frequency of CHIKV Carib DVG arising in each replicate in 2 mammalian cell lines (Vero, Huh7) and 2 mosquito cell lines (Aag2, U4.4), determined after RNA deep-sequencing of each sample and quantification of DVG with Bbmap pipeline output. Samples with coverage under 200 were excluded from analysis. DVG count is represented in log scale (y axis) at each passage (x axis). (**B**) CHIKV Carib or IOL DVG heat maps in different cell types. The viral population of each passage was deep sequenced (RNAseq) and analyzed through BBmap pipeline. An average of all passages and replicates of the normalized frequency of each deleted nucleotide position (x axis) throughout the full genome is shown as a heat map (shades of orange), for each different cell type (y axis). (**C**) Analysis of the deletions generated by high MOI passages by CHIKV Carib. Start (x axis) and stop (y axis) positions of the breakpoint of DVGs generated are plotted for each cell type. The different clusters are called A, B, C, D.

In order to capture DVGs containing deletions as they arise in cell culture, the Caribbean strain of CHIKV (CHIKV Carib) was serially passaged in triplicate at high MOI in mammalian (Vero, Huh7) and mosquito (Aag2, U4.4) cells. RNA was extracted from the clarified supernatant of each replicate at each passage and RNA deep sequencing was performed to identify DVGs. As expected, the amount of DVGs between the first and last passages increased by between 1 and 5 orders of magnitude in all cell types, and the rates and oscillations differed with each cell type (Fig 1A).

### Characterization of the DVGs generated *in vitro*

Next, we pooled the data from each cell line to identify potential deletion hotspots in the different cell types. The resulting heat map (Fig 1B) reveals the frequency at which each nucleotide position was deleted (regardless of the total length of the deletion), where the x-axis indicates the nucleotide position along the full CHIKV genome. Each row represents a different cell type. We observed clear differences in regions with higher deletion frequencies between cell types, suggesting that the same viral strain generates different deletion variants depending on the host environment. In Vero and Huh7 cells, deletions were abundant in the region spanning the non-structural proteins nsP1-nsP2, yet the hotspot profile was shorter in HuH7 compared to Vero cells. In Aag2 cells, deletions were more widely spread across the genome. Contrary to the other cell lines, where deletions occurred in the first half of the CHIKV genome, DVGs generated in U4.4 cells more often exhibited deletions in the second half of the genome (from nucleotides 6000 [nsP3] to 11500 [3’UTR]). Of note, the same experiment with the CHIKV IOL (Indian Ocean Lineage) strain had similar deletion profiles as the CHIKV Carib strain in Vero and Aag2 cells. However, CHIKV IOL deletions differed strongly in Huh7 (where the strongest hotspot is from nucleotides 9000 [E2] to 11000 [3’UTR]) and in U4.4 cells (whose profile, in this case, was similar to the profile obtained in Aag2 cells) (Fig 1B). Taken together, these results suggest that both the virus strain, as well as the cellular environment, influence the generation and maintenance of DVGs during passaging *in vitro*.

To visualize the different DVGs in these viral populations, the specific start (*x* axis) and stop (*y* axis) breakpoints of individual DVGs with deletions of >100 nucleotides were plotted (Fig 1C). The analysis revealed predominant DVGs forming three clusters (cluster A, B and C) based on the location and the size of the deletion that was observed in all cell types, while a fourth cluster (cluster D) was more prominent in mosquito cells. Cluster A presented relatively small deletions of approximately 2500 nt, between the nsP1 and nsP2 genes. Cluster C presented large deletions of over 9000 nt from the end of nsP2 to the beginning of the 3’UTR, and for cluster B, an even larger deletion spanning nsP1 to the 3’UTR. In *Aedes* mosquito cells (Aag2 and U4.4), another cluster D was present with deletions of approximately 5000nt between nsP3 and the 3’UTR.

### Generation of chikungunya DVGs in mosquitoes *in vivo*

**Fig 2:**
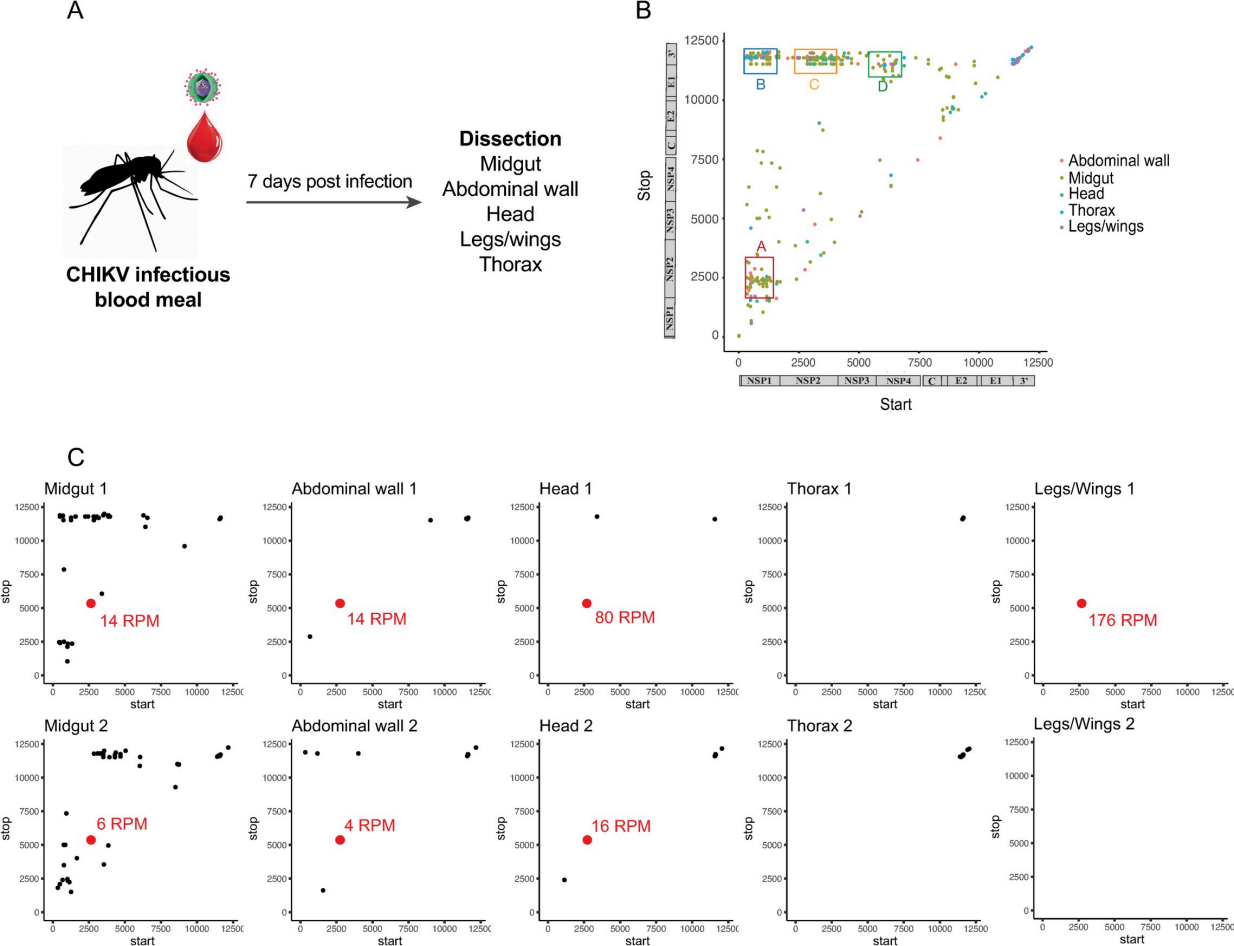
Generation of chikungunya DVGs in mosquitoes *in vivo*. (**A**) *Aedes aegypti* mosquitoes were fed a blood meal infected with 10^6^ PFU/ml of CHIKV Carib. After 7 days, ten mosquitoes were sacrificed and midgut, abdominal wall (body), thorax, head and legs/wings were dissected. After verification of infection by titration, viral RNA was extracted and deep-sequenced. (**B**) Analysis of the deletions generated by CHIKV Carib in nine infected mosquitoes pooled together. Start (x axis) and stop (y axis) positions of the breakpoint of DVGs generated are plotted for each organs. (**C**) Start (x axis) and stop (y axis) positions of the breakpoint of DVGs generated in the different organs of 2 individual mosquitoes. The red dots represent the deletion starting at 2695 and ending at 5358, this DVG is called CM1. The number next to it is the number of read per million reads (RPM).

To see if DVGs were also generated during *in vivo* mosquito infection, we fed *Aedes aegypti* mosquitoes an infected blood meal containing 10^6^ PFU/ml of the Caribbean strain of CHIKV. After allowing the infection to disseminate throughout the mosquitoes over seven days, we sacrificed and dissected ten of them to collect individual organs: midgut, abdominal wall (body), thorax, heads and legs/wings (Fig 2A). RNA was extracted and sequenced, and the data were analyzed using the BBmap pipeline. Similar DVG patterns to the data generated in Aag2 cells (Fig 1C) were observed, with four distinct clusters (Fig 2B). One mosquito was not infected. In two mosquitoes out of the nine remaining (Fig 2C), we found the same DVG (named CM1) in several organs (midgut, body, head and legs/wings for one mosquito and midgut, body and head for the other mosquito). No other mosquitoes, nor the viral stock used to infect them, contained this DVG-CM1. These results indicate that DVGs are readily generated in the mosquito host, and that they share similar deletion profiles as what was observed in cell culture.

### Chikungunya DVG candidates are non replicative

In the previous sections we uncovered all of the possible DVGs generated during virus infection. Our goal was to down-select, from the hundreds of individual DVGs, those that would most likely and efficiently compete with wild-type virus. In other words, to identify which of the total DVGs would be the most potent defective interfering particles. Our rationale was that such DVGs would occur more frequently at high MOI passage, and would increase to higher frequency over time as the DVG competes with wildtype virus for resources. Thus, we selected DVGs based on: having high frequency, being maintained throughout the passage series and occurring in several replicates.

**Fig 3:**
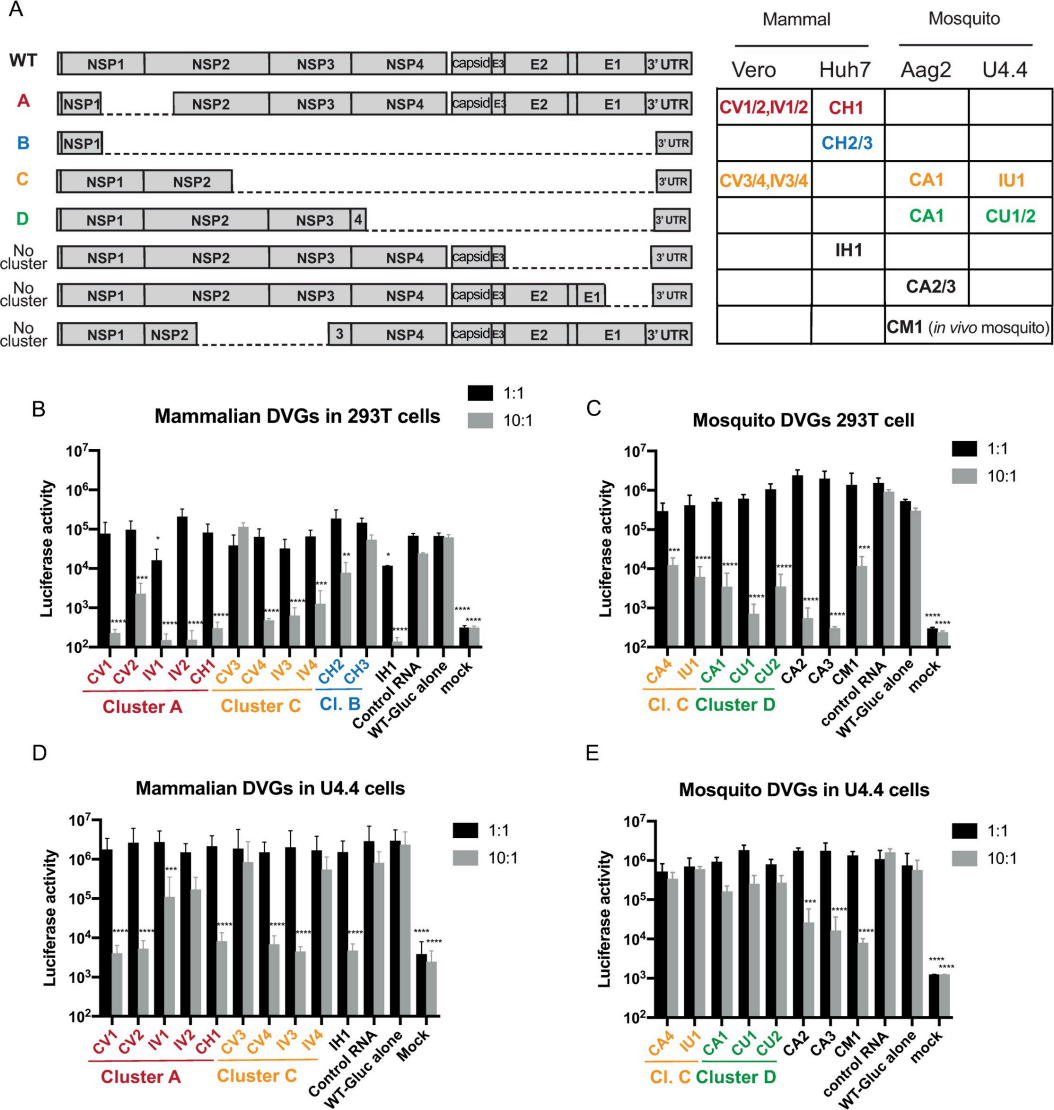
Defective viral genomes can interfere with chikungunya virus replication. (**A**) Schematic of the DVG candidates and the cell type and strain from which they were derived (C Carib, I IOL). (**B-E**) Measuring DVG activity *in vitro*. *In vitro* transcription of each DVG candidate (identified from mammalian cell passage (**B,D**) or mosquito cell passage (**C,E**)) or of a control RNA was co-transfected with CHIKV Carib-luciferase at 1:1 or 10:1 molar ratio in 293T cells (**B,C**) or U4.4 cells (**D,E**). After 48 hours, luciferase activity was measured. (Representative result of one experiment out of three independent experiments is shown, n = 3, *p<0,05, **p<0,01, ***p<0,001, ****p<0,0001, ns non significant compared to wild-type after Dunnett’s multiple comparisons on one-way ANOVA test).

In addition to DVG-CM1, carrying a deletion from nucleotide 2695 (middle of nsP2) to nucleotide 5358 (end of nsP3), we selected and cloned another 19 DVGs, from the cell passage deep sequencing data of CHIKV Carib (described above) and CHIKV IOL, along with representatives from all four clusters (Fig 3A). We named DVGs according to their virus strain of origin (C for CHIKV Carib and I for CHIKV IOL) and the cell in which they were generated (V for Vero, H for Huh7, A for Aag2 and U for U4.4, M for *in vivo* mosquito). Their genomic composition is shown in Fig 3A and their exact deletions are listed in Table 1. Three additional candidates did not belong to any defined cluster, but were present at high frequency in several replicates and were maintained throughout passages (Table 1).

**Table 1:** List and characteristics of chikungunya virus DVGs in our study.

DVG	Strain^a^	Host^b^	start^c^	Stop^d^	deletion length	DVG length	Deletion location^e^
IV1	IOL	Vero	435	3123	2689	9163	NSP1-2
IV2	IOL	Vero	663	2878	2216	9636	NSP1-2
IV3	IOL	Vero	3892	11508	7617	4235	NSP2-3'UTR
IV4	IOL	Vero	3850	11629	7780	4072	NSP2-3'UTR
IH1	IOL	Huh7	8495	11593	3099	8753	E3-3'UTR
IU1	IOL	U4.4	3570	11509	7940	3912	NSP2-3'UTR
CV1	Carib	Vero	435	3123	2689	9541	NSP1-2
CV2	Carib	Vero	663	2878	2216	10014	NSP1-2
CV3	Carib	Vero	3893	11527	7635	4595	NSP2-3'UTR
CV4	Carib	Vero	3893	11704	7812	4418	NSP2-3'UTR
CH1	Carib	Huh7	508	2188	1681	10549	NSP1-2
CH2	Carib	Huh7	555	11501	10947	1283	NSP1-3'UTR
CH3	Carib	Huh7	555	11678	11124	1106	NSP1-3'UTR
CU1	Carib	U4.4	5628	11468	5841	6389	NSP3-3'UTR
CU2	Carib	U4.4	5897	11528	5632	6598	NSP3-3'UTR
CA1	Carib	Aag2	6183	11784	5602	6628	NSP4-3'UTR
CA2	Carib	Aag2	3050	6141	3092	9138	NSP2-4
CA3	Carib	Aag2	373	2344	1972	10258	NSP1-2
CA4	Carib	Aag2	2698	11790	9093	3137	NSP2-3'UTR
CM1	Carib	mosquito	2695	5358	2664	9566	NSP2-3

a Virus strain of origin

b host cell type in which DVG was identified

c nucleotide position where the deletion starts

d nucleotide position where the deletion stops

e genes and genomic regions where deletion occurs; DVG, defective viral genomes; Deletion location, the region of the genome affected by the deletion that may include envelope proteins (E), nonstructural proteins (NSP) or 3’untranslated region (3’UTR).

To confirm that the selected DVGs were indeed defective genomes, unable to self-replicate in absence of virus, we transfected *in vitro* transcribed RNA of each DVG or wild-type virus in 293T cells. We harvested each DVG- or control-transfected cells at 8, 20, 28, and 44 hours post transfection, extracted RNA from the cells and performed an RT-qPCR. Contrary to CHIKV Carib full-length virus RNA that had an increasing RNA copy number throughout the timepoints, all DVG RNA copy numbers decreased over time, reflecting the progressive degradation of transfected RNA, and demonstrating that none of the candidate DVGs could self-replicate (S1 Fig).

### Chikungunya DVGs interfere with wild-type virus *in vitro*

We next tested the interfering activity of the 20 DVGs derived from either mammalian cell culture (Fig 3B) or mosquito host environments (Fig 3C) in mammalian cells. We chose 293T cells for their high transfectability, and a CHIKV Carib virus expressing Gaussia luciferase under a sub-genomic promoter (CHIKV Carib-Gluc) as the target virus for rapid quantification. To test the DVGs that were derived from mammalian or mosquito cell culture, 293T cells were transfected with a mix of *in vitro* transcribed RNA corresponding to one DVG of interest (or a control RNA) and the CHIKV Carib-Gluc full length virus RNA at a 1:1 or 10:1 molar ratio. Supernatants were harvested at 48 hours to measure luciferase activity as a surrogate measure for wild-type virus replication, since titers and luminescence correlated (S2 Fig). At a 1:1 molar ratio, most DVGs did not reduce wild-type virus luciferase expression, but transfection of IV1 and IH1 showed a modest decrease (p< 0,05). However, increasing the amount of DVG RNA to 10 times that of CHIKV Carib-Gluc RNA significantly inhibited virus replication in nearly every case by 1 to 3 orders of magnitude (Fig 3B and 3C). Of note, the smallest DVGs with the largest deletions (CH2 and CH3 from cluster B) had no, or little, interference activity at the highest DVG:CHIKV Carib-Gluc molar ratio, with a decrease of less than 1 log (Fig 3B). These results show that DVGs derived from both mammalian or mosquito cell culture can inhibit virus replication in mammalian cells when introduced exogenously.

To determine whether these mammalian and mosquito cell-derived DVGs could also inhibit virus replication in mosquito cells, we repeated the experiment in *Aedes albopictus* cells (U4.4). As for 293T cells, no interference was observed at a 1:1 molar ratio; but considerable inhibition with decreases of 1 to 2 log of luciferase activity was observed at 10:1 ratios for all of the DVGs that previously inhibited virus in mammalian cells, except for IV4. As seen in 293T cells, no interference was observed for DVG CV3 in mosquito cells (p>0,05) (Fig 3D), which along with CH3, were the only two of 20 DVG candidates to fail to inhibit virus in any condition. On the other hand, most of the mosquito cell-derived DVGs that could inhibit virus in 293T cells completely lost their interference activity in mosquito cells; only DVG CA2, CA3 and CM1 significantly reduced luciferase activity by 1 to 2 log in U4.4 cells at a molar ratio of 10:1 (Fig 3E).

### Chikungunya DVGs can be broad-spectrum inhibitors

**Fig 4:**
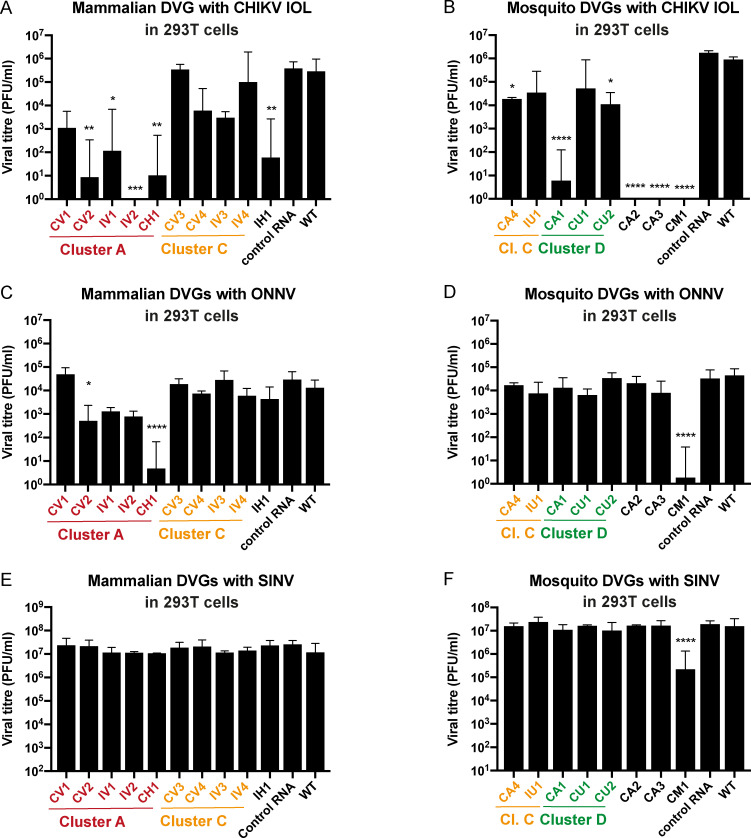
Defective viral genomes have broad-spectrum inhibiting activity in the alphavirus family. The DVGs identified in mammalian cell passage (**A,C,E**) or mosquito cell passage (**B,D,F**) were tested for inhibition of CHIKV IOL (**A, B**), O’nyong-nyong virus (ONNV) (**C, D**) and Sindbis virus (SINV) (**E, F**). *In vitro* transcribed DVG candidates were co-transfected with CHIKV IOL (**A,B**), ONNV (**C,D**) and SINV (**E,F**) at 10:1 molar ratio in 293T cells. After 48 hours, viral titer was measured. (Representative result of one experiment out of three, n = 3, *p<0,05, **p<0,01, ***p<0,001, ****p<0,0001, ns non-significant compared to wild-type after Dunnett’s multiple comparisons on one-way ANOVA test).

The CHIKV Indian Ocean lineage belongs to the East, South and Central African (ECSA) genotype and has approximately 7% nucleotide divergence with the CHIKV Caribbean strain that belongs to the Asian genotype[17]. In the previous experiments, DVGs derived from either the Indian Ocean Lineage or Caribbean strains were tested against the Caribbean strain virus expressing Gaussia luciferase. Importantly, all of the CHIKV IOL-derived DVGs inhibited the Caribbean strain, showing that DVGs can act broadly within the same virus species (Fig 3B–3E). To extend these results, we repeated the same experiment in 293T using CHIKV IOL strain as the target virus. In this case, we measured inhibition by virus titer instead of luminescence. Most DVGs resulted in reduced virus titers, either modestly or significantly, whether they were identified from mammalian (Fig 4A) or mosquito (Fig 4B) environments.

We next investigated whether CHIKV DVGs could inhibit even more broadly within the alphavirus genus. We carried out the same experiment in 293T cells with O’nyong’nyong virus (ONNV, CHIKV’s closest relative) or Sindbis virus (SINV, a very distant relative) as the targets. While the inhibitory effect was lost for most DVGs, some DVGs still significantly inhibited O’nyong’nyong virus (CV2 and CH1) (Fig 4C). Of the mosquito-derived DVGs, CM1 maintained inhibitory activity with close to no detectable O’nyong’nyong virus titers (Fig 4D). When tested against the distantly related Sindbis virus, interference activity was lost for all DVGs, but for CM1 that inhibited Sindbis virus by 2 logs (Fig 4E and 4F). These results confirm that some DVGs derived from one alphavirus can have inhibitory activity on different strains, including closely, and in some cases more distantly related viruses from the same genus.

### Chikungunya DVGs block viral dissemination *in vivo*, in *Aedes aegypti* mosquitoes

**Fig 5:**
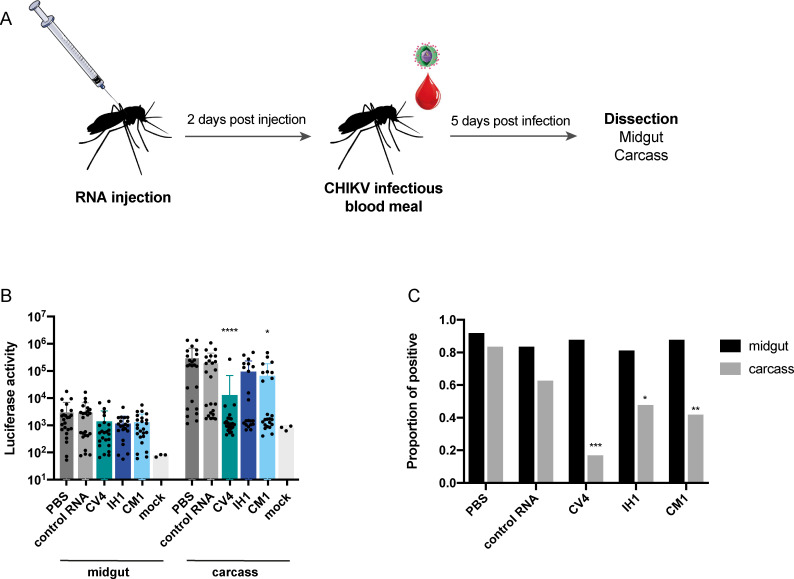
Defective viral genomes prevent viral dissemination of chikungunya virus in *Aedes aegypti* mosquitoes. (**A**) 150 ng of DVG candidate RNA (IH1, CV4 or CM1), a control RNA or PBS were injected into *Aedes aegypti* mosquitoes 2 days prior to being fed an infected blood meal containing 10^6^ PFU/ml. After 5 days, mosquitoes were sacrificed, and midguts were separated from carcasses. (**B**) Luciferase activity of midgut or carcass of each mosquito was measured. (**C**) After virus titration, the proportions of positive midguts or carcasses (PFU≥100 PFU/ml) were determined. (Representative result of one experiment out of two independent experiments is shown, n = 24, *p<0,05, **p<0,01, ***p<0,001, ns non significant compared to PBS after one-way ANOVA with multiple comparison (B) or Chi square test, multiple comparison with Holm correction (C)).

After demonstrating that DVGs can be used to limit/inhibit infection *in vitro*, we tested if their interference activity could be used to block infection or dissemination in the mosquito host. To do so, we injected purified RNA of the CV4, IH1 or CM1 DVGs, a control RNA, or PBS into *Aedes aegypti* mosquitoes 2 days prior to feeding them with a blood meal containing the CHIKV Carib-Gluc virus. Five days post infection, mosquitoes were sacrificed and midguts were dissected from the rest of the carcass (Fig 5A). Virus replication was measured in each mosquito by quantifying luciferase activity. Replication in the midgut was similar in all mosquitoes regardless of whether interfering DVG candidates were present or not. However, replication was significantly reduced in CV4- and CM1-treated mosquito carcasses (Fig 5B). Virus in midguts (representing infection) and carcass (a proxy for viral dissemination) was then quantified, and classified as positive or negative, depending on whether infectious viruses were detected or not (limit of detection 30 PFU per organ). All groups had a similar proportion of positive midguts (between 81 and 91,7%) (Fig 5C), confirming that the number of infected mosquitoes following bloodmeal feeding was similar for each group. On the other hand, virus dissemination was significantly impacted by the presence of DVGs: mosquitoes injected with either CV4, IH1 or CM1 DVGs had lower dissemination rates compared to PBS injected mosquitoes (16,7%, 47,6% and 41,7% of total infected mosquitoes in each group) (Fig 5C). These results confirm that DVGs can impact viral replication and dissemination *in vivo*, in the mosquito host.

## Discussion

Described in all RNA virus families[5, 6] and often considered a waste product of viral replication, defective interfering particles, and more broadly speaking DVGs, have recently garnered attention for their possible use as antivirals [6, 15, 18–22]. Indeed, the presence of DVGs in natural human infections correlates with milder disease in clinical studies on respiratory syncytial virus and influenza virus[10, 13]; and influenza defective interfering particles protect mice against lethal challenge[18, 19, 23]. In this study, we aimed to characterize, as widely as possible, the DVGs bearing deletions that arise during CHIKV infection *in vitro* in mammalian and mosquito environments and *in vivo* in its mosquito host. We show that chikungunya DVGs arise in all environments, *in vitro* and *in* vivo, but their type and abundance can depend on the host and cell type, and on the virus strain, highlighting that both cellular environment and the viral genome bear determinants of DVG generation. For example, cluster D was strongly represented in both *Aedes spp*. mosquito cells, even though the deletion did not occur at the exact same position, but it was rare in mammalian cells (Fig 1C).

To find DVGs that could be useful as antivirals, we selected the most redundant DVGs, that arose in several conditions and were propagated over passages, which we hypothesized to be indicative of higher fitness and the ability to be packaged by wild-type virus, which are important characteristics of interfering DVGs. When used in higher quantity relative to wild-type virus, most candidates showed promising inhibiting capacity. However, our analysis could not identify a single or clear reason why one DVG candidate was better than another. Of note, because smaller genomes should be replicated more quickly[7, 8], the conventional dogma proposes that the smallest DVGs would most efficiently hijack the parental virus replication machinery and outcompete wild-type virus. However, in our work, the smallest DVGs identified (two DVGs from cluster B) had no or very little interference activity. This observation implies that strict competition for the replicase complex and replication speed is not the only mechanism by which DVGs interfere with parental virus. It is possible that the DVGs described here also have immunostimulatory activity as has been described for other viruses[7, 9–11, 24–29]. A specific sequence or RNA structure might render one DVG more immunostimulatory. Additionally, they may more effectively compete for structural proteins or host ressources[8, 30].

Of the DVGs generated in *Aedes aegypti in vivo*, CM1 attracted our attention because it could cross bottlenecks. During a natural viral infection, after an infectious blood meal, the midgut is the first organ infected before the virus reaches the hemocoel to disseminate to all other organs[31]. Previous work has shown that virus exit from the midgut is the main population bottleneck during mosquito infection with a drastic reduction in population size, followed by egress from the salivary glands, a mandatory step for viral transmission to the mammalian host through infected saliva[31–35]. CM1 was newly generated in the midguts of two independent mosquitoes and found to disseminate to the body wall, head and legs/wings of these same mosquitoes. The possibility that CM1 was generated *de novo* in other organs is unlikely because this DVG did not appear in any organs of any other mosquitoes that had not generated it in the midgut. It is not clear how or why CM1 is able to cross the midgut bottleneck. One possibility is that it belongs to a collective infectious viral unit, a structure that simultaneously contains and transports multiple viral genomes to a single cell, such as polyploid virions, aggregates of virions or virion-containing lipids vesicles[36, 37]. The possibility of collective infectious unit containing DVGs has already been proposed. Indeed, Leeks *et al*. modeled that if the viral population contains many DVGs, the collective infectious unit could become very large. Nonetheless, the presence of interfering DVGs would disfavor transmission within the collective infectious unit compared to free virions[38]. While CM1 had an inhibiting activity at high molar ratio compared to the parental virus, its frequency in mosquito samples was very low (4 to 176 reads per million).

A valuable characteristic of some of these interfering DVGs is their broad-spectrum activity, with inhibition not only on other CHIKV strains but also on other alphaviruses. This cross reactivity had already been reported with influenza and Sendai virus, since their DVGs were shown to be efficient vaccines or vaccine adjuvants in mammalian models not only against the virus from they were derived, but also against unrelated viruses[15, 18–22]. From a therapeutic point of view, this is of particular interest since the risk of worldwide dissemination of arthritogenic alphaviruses is well accepted[3, 39–43], especially since no antivirals or vaccines against any of these viruses are currently licensed. In this context, any treatment (antiviral or vaccine) that could work across a viral genus or family would be helpful in facing outbreaks to come.

It will be important in the future to test if chikungunya DVGs could function as antivirals in mammalian *in vivo* models as well; however, a proper delivery system still needs to be developed. In previous work on influenza A and Sendai DVGs, authors isolated DVGs by ultra-centrifugation of a mix of wildtype virus and DVG on sucrose gradients[15, 18–22]. However, we could not successfully separate chikungunya DVGs from wild-type virus using these methods. New approaches, such as delivering RNA or DNA in nanoparticles or nanostructured lipids[44, 45], or attempting to package DVGs in virus-like particles (VLPs) could be explored. Another major hurdle to overcome is the choice of *in vivo* model. Wild-type C57BL/6 could be used, but as mice develop only a transient, mild infection, it would be difficult to demonstrate an inhibitory effect for even the best DVG candidates. While the most commonly used mouse models rely on interferon α/β knockouts[46, 47], the induction of innate immunity would be excluded, which may be an important mechanism of action in chikungunya DVGs [10, 24–27, 29].

Finally, when injected in *Aedes aegypti* mosquitoes two days before an infectious blood meal, three DVGs significantly reduced viral dissemination from the mosquito midgut, thereby shifting the status of mosquitoes from being competent to incompetent vectors for CHIKV spread. This result is a proof of principle that DVGs might be useful tools in controlling CHIKV infection in the vector population. As releasing incompetent mosquitoes in the wild during an arbovirus outbreak has already been shown to be an effective epidemic control strategy[48, 49], and despite the need for a simpler delivery system, it is tempting to propose that interfering DVGs could be used as a control strategy not only for CHIKV, but for any arbovirus. Furthermore, interfering DVGs are presumably safe antiviral tools because they are inert molecules that cannot self-replicate, and are only active when wild-type virus is present.

In conclusion, this work describes the different types of deleted DVGs generated during CHIKV infection in both vertebrate and invertebrate environments *in vitro* and *in vivo* in the mosquito vector. Moreover, we identified criteria to down-select the best defective interfering particle candidates able to inhibit wild-type CHIKV *in vitro* in both vertebrate and invertebrate hosts. An interesting observation is the broad-spectrum activity of some interfering DVGs able to interact with related alphaviruses. Finally, we show that pre-exposure to a DVG can modulate viral dissemination in mosquitoes *in vivo*. These results strengthen the idea that defective interfering particles might be a useful therapeutic tool for CHIKV infection as well as an efficient vector control strategy.

## Material and methods

### Ethics statement

Human blood used to feed mosquitoes was obtained from healthy volunteer donors who gave formal written consent at the ICAReB biobanking platform (BB-0033-00062/ICAReB platform/Institut Pasteur, Paris/BBMRI AO203/[BIORESOURCE]) of the Institut Pasteur, under the CoSImmGen and Diagmicoll protocols which were approved by the French Ethical Committee (CPP) Ile-de-France I. The Diagmicoll protocol was declared to the French Research Ministry under the reference: DC 2008–68 COL 1.

### Cells

Vero, Huh7, 293T and BHK cells were grown in Dulbecco’s modified Eagle’s medium (DMEM), containing 10% fetal calf serum (FCS), 1% penicillin/streptomycin (P/S; Thermo Fisher) and 1% non essential amino-acid (Thermo Fisher) in a humidified atmosphere at 37°C with 5% CO_2_. U4.4 and Aag2 cells were maintained in Leibovitz's L-15 medium with 10% FCS, 1% P/S, 1% non-essential amino acids (Sigma) and 1% tryptose phosphate (Sigma) at 28° C.

### Virus

The viral stocks were generated from CHIKV infectious clones derived from the Indian Ocean lineage, ECSA genotype (IOL; described in [50]) or from the Caribbean strain, Asian genotype (Carib; described in [51]). To test DVG interference activity, a CHIKV infectious clone containing the Gaussia luciferase gene under a subgenomic promoter was used (obtained from Andres Merits). *In vitro* transcription (IVT) with SP6 mMESSAGE mMACHINE kit (Invitrogen) was performed on *Not I* linearised plasmids prior to transfection in BHK using lipofectamine 2000 (Invitrogen). After one passage in Vero cells, the stocks were titered and kept at -80°C before use.

The O'nyong’nyong virus (ONNV) infectious clone, under an SP6 promoter, was obtained from Andres Merits. Sindbis virus was generated from the pTR339 infectious clone described in [16].

### Plaque assay

Viral titration was performed on confluent Vero cells plated in 24-well plates, 24 hours before infection. Ten-fold dilutions were performed in DMEM alone and transferred onto Vero cells. After allowing infection, DMEM with 2% FCS, 1% P/S and 0,8% agarose was added on top of cells. Three days post infection, cells were fixed with 4% formalin (Sigma), and plaques were manually counted after staining with 0,2% crystal violet (Sigma).

### Viral passages

Cells were seeded in 24-well plates to reach approximately 80% confluency the next day. For passage 1, virus was diluted in PBS to obtain a multiplicity of infection of 5 PFU/cell (high MOI). Cells were incubated with the viral inoculum at 37°C for 1 hour. Following virus adsorption, the inoculum was removed, the infected cells were washed with PBS, and replaced with the appropriate cell culture medium containing 2% (v/v) FCS. At 48–72 hour post infection, supernatant was harvested and clarified by centrifugation (12 000 x g, 5 min). The following passages were performed blindly, using a high volume (300 μl) of the clarified supernatant from previous passage to infect naïve cells followed by the same procedure. A total of 10 passages were performed. Each passage was titered by plaque assay to determine at which passages DVG accumulation may have resulted in interference. At least three replicates were performed per cell type.

### Sequencing

RNA of 100 ul of each sample supernatant was extracted using TRIzol reagent (Invitrogen) or ZR viral RNA kit (Zymo) following the manufacturer’s protocol. RNA was eluted in 15–30 μL nuclease-free water. After quantification using Quant-iT RNA assay kit (Thermo Fisher Scientific), RNA libraries were prepared with NEBNext Ultra II RNA Library kit (Illumina). Multiplex oligos (Illumina) were used during library preparation. The quality of the libraries was verified using a High Sensitivity DNA Chip (Agilent) and quantified using the Quant-iT DNA assay kit (Thermo Fisher Scientific). Sequencing of the libraries (diluted to 1 nM) was performed on a NextSeq sequencer (Illumina) with a NextSeq 500 Mid Output kit v2 (Illumina) (151 cycles).

### Next generation sequencing data analysis

All analyses were performed using BBTools suite (Bushnell B.—sourceforge.net/projects/bbmap/). First, fastq files generated from sample sequencing were trimmed for low-quality bases and adaptors using BBDuk. Then, alignment was performed using BBmap and the appropriate CHIKV reference sequence (Carib—GenBank accession no. LN898104.1, IOL—GenBank accession no. AM258994). BBMap’s variant caller CallVariants was used to report deletion events, and the overall DVG frequency per sample was calculated as the sum of the number of junction read counts (n) corresponding to each DVG and normalised as 5/3 × n/N, where N denotes the 98th percentile of the coverage per position. Multiplying by 5/3 aims to correct for the detection limit. Indeed, even if reads are 150 nucleotides long, aligned portions are usually only 30 to 120 nucleotides long. This implies that a read starting 30 or less nucleotides upstream of the breakpoint will be aligned on the right side but not on the left side of the breakpoint, and thus, will not be considered as a DVG. Consequently, we are missing (30+30)/150 = 60/150 = 2/5 of the deletions, and the counts must be multiplied by 5/3 as a correction factor. Heatmaps illustrate the deletion score per nucleotide position based on deletion events removing that particular position. Specifically, scores were computed as the sum of the number of reads per million reads (RPM) supporting the deletion of a specific nucleotide position. For plotting start/stop breakpoints, deletions with lengths below 10 nucleotides were discarded.

### Cloning selected defective genomes

Defective genomes selected from passages were cloned in the CHIKV infectious clones (under SP6 promoter) corresponding to their strain, using the previously described In Vivo Assembly (IVA) method[52] or using In-Fusion reagent (Takara Bio Reagent). Primers were designed using SnapGene software, with a melting temperature of 60°C, and obtained from Integrated DNA Technology (IDT). 50 μl PCR using either Phusion high fidelity DNA polymerase (Thermo Fisher) or Q5 DNA polymerase (NEB) was carried out with a melting temperature of 57°C for Phusion enzyme or 65°C for Q5 polymerase. 18 cycles were carried out and the PCR products were then *Dpn I* (Thermo Fisher)-treated for at least 2 hours to remove the plasmid template and purified (Macherey Nagel PCR and gel purification kit). When IVA technique was used, 2ul of the PCR products were directly transformed in XL10-Gold extra competent cells (Stratagene) according to supplied protocol. When In-Fusion reaction was needed, PCR products were ligated using In-Fusion reagent (Takara Bio Reagent) following supplier instructions and 1ul was transformed in Turbo cells (NEB).

### Luciferase assay to test DVG interference activity

IVTs were performed using the SP6 mMESSAGE mMACHINE kit (Invitrogen) from *Not I* linearized infectious clones of DVG and CHIKV Carib-GLuc (see above). The internal control of the IVT kit (pTRI-Xef) was also *in vitro* transcribed and used as the control RNA in all experiments. RNA production was quantified by Qubit RNA TS Assay kit (Thermo Fisher) and diluted to be at the indicated molar ratios compared to the Carib-GLuc CHIKV. Mixed DVG and full genome RNA were transfected in 293T or U4.4 cells seeded in 96-well plates with TransIT-mRNA transfection kit (Mirus) following the supplier’s protocol (25 ng of Carib-GLuc CHIKV per well). The medium was changed 4 hours post transfection to avoid cellular toxicity. 48 hours after transfection, supernatant was collected and mixed with coelenterazine (supplied by Y. Janin, Institut Pasteur) at a final concentration of 0,05μM and luminescence was measured on a Tecan Infinite 200 microplate reader.

When tested with non-luciferase expressing viruses, the procedure was followed the same way but the supernatant was titered by plaque assay 48 hours post transfection.

### RT-qPCR to test for DVG self-replication

50ng of DVG or CHIKV Carib IVT was transfected in 293T cells (seeded the day before in 48-well plates), in triplicate, as described above. 8 hours post transfection, supernatant was removed, and cells were washed 3 times with PBS before adding fresh medium. At 8, 20, 28 and 44 hours post-transfection, 200ul of lysis buffer from ZR 96 viral RNA kit (Zymo) was added on the cells after supernatant removal and stored at -20°C until all time points were collected. Cellular RNA was extracted with the ZR 96 viral RNA kit and eluted in 15 μl.

TaqMan RNA-to-Ct One-step RT-PCR kit (Applied Biosystems) was used to perform a quantitative RT-PCR spanning the 5’UTR-nsP1 region, with 5’-GAGACACACGTAGCCTACCA-3’ as the forward primer, 5’-GGTTCCACCTCAAACATGGG-3’ as the reverse, and 5’- [6-FAM] ACGCACGTTGCAGGGCCTTCA-3’ as the probe. After 20min at 50°, and 10 min at 95°, 40 cycles were performed (95°C for 15 seconds followed by 60°C for 1 minute).

### Mosquitoes

*Aedes aegypti* female mosquitoes belonging to the 7th generation from wild mosquitoes collected in Kamphaeng Phet Province (Thailand) were grown at 28°C, 70% relative humidity and a 12 hour light: 12 hour dark cycle, and fed with 10% sucrose.

### Mosquito infections

A day before blood feeding, 6 to 10-day old females were selected and starved for a day. A blood meal containing 10^6^ PFU/ml of CHIKV was offered to a pool of 60 females through a membrane feeding system (Hemotek Ltd) set at 37°C with a piece of desalted pig intestine as the membrane. Following the blood meal, fully engorged females were selected and incubated at 28°C, 70% relative humidity and under a 12 hour light: 12 hour dark cycle with permanent access to 10% sucrose.

After 7 days, the mosquitoes were sacrificed and dissected to collect heads, thorax, midgut, legs and wings (legs/wings), and abdominal wall (body). Each organ was ground in 300 ul of L15 supplemented with 2% FBS with Qiagen TissuLyser 2 machine, then clarified by centrifugation before titration and RNA extraction with TriZol reagent for RNA deep sequencing.

### Mosquito injections

One hundred and fifty nanograms of RNA produced by *in vitro* transcription (as described above) and purified by phenol-chloroform was mixed with Leibovitz's L-15 medium and Cellfectin II Reagent (Thermo Fisher) following the supplier’s instructions. For each condition, forty 6 to 8 day-old females were injected intra-thoracically with 300nl of this mix with Nanoject III Nanoliter Injector (Drummond scientific company). Two days later, after a night of starvation, mosquitoes were fed with an infectious blood meal of 10^6^ PFU/ml of CHIKV Carib-GLuc as described above. After 5 days, mosquitoes were sacrificed; midgut and carcass were dissected and ground as mentioned before. Each sample was then tested for luciferase activity and titered by plaque assay.

## Supporting information

S1 Fig Chikungunya virus DVGs are not self-replicating. 293T cells were transfected with DVG or CHIKV Carib *in vitro* transcribed RNA and harvested at 8, 20, 28 and 44 hours post transfection. Cellular RNA was extracted and used to perform a RT-qPCR with a Taqman probe.
(EPS)

S2 Fig Measuring DVG activity *in vitro* by virus titration. *In vitro* transcribed RNA of each DVG candidate (derived from mosquito cells) was co-transfected with CHIKV Carib-luciferase RNA at 1:1 or 10:1 molar ratio in U4.4 cells. After 48 hours, samples were titered and luciferase activity was measured (Fig 3E). (n = 3, **p<0,01, ****p<0,0001, ns non-significant compared to wild-type after Dunnett’s multiple comparisons on one-way ANOVA test).
(EPS)
